# Supplementary material for: Mutations in the tomato gibberellin receptors suppress xylem proliferation and reduce water loss under water-deficit conditions
Source: J Exp Bot. 2020 Mar 16;71(12):3603–12. doi: 10.1093/jxb/eraa137 (PMC7475260; doi:10.1093/jxb/eraa137)
Supplement: eraa137_suppl_Supplementary_Table_Figure [file eraa137_suppl_supplementary_table_figure.pdf]

Supplementary Figures

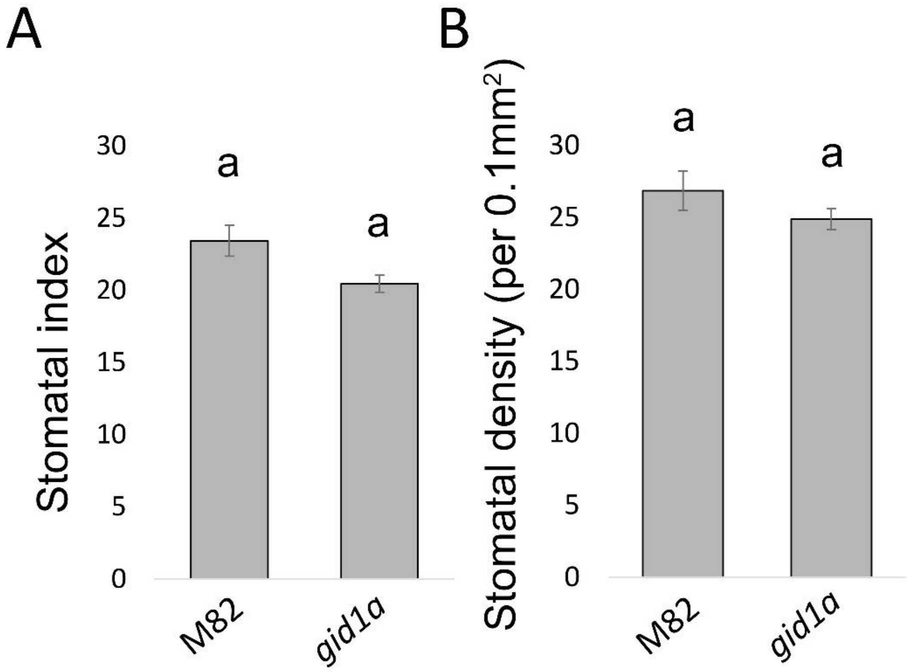

Fig. S1. The loss of GID1a has no effect on stomatal density or index. **A.** Stomatal index (number of stomata to epidermal cells). **B**. Stomatal density (per 0.1 mm^2). Values are means of five replicates (5 different plants) ± SE. Letters above the columns represent significant differences between respective lines (Student's t test, P<0.05).

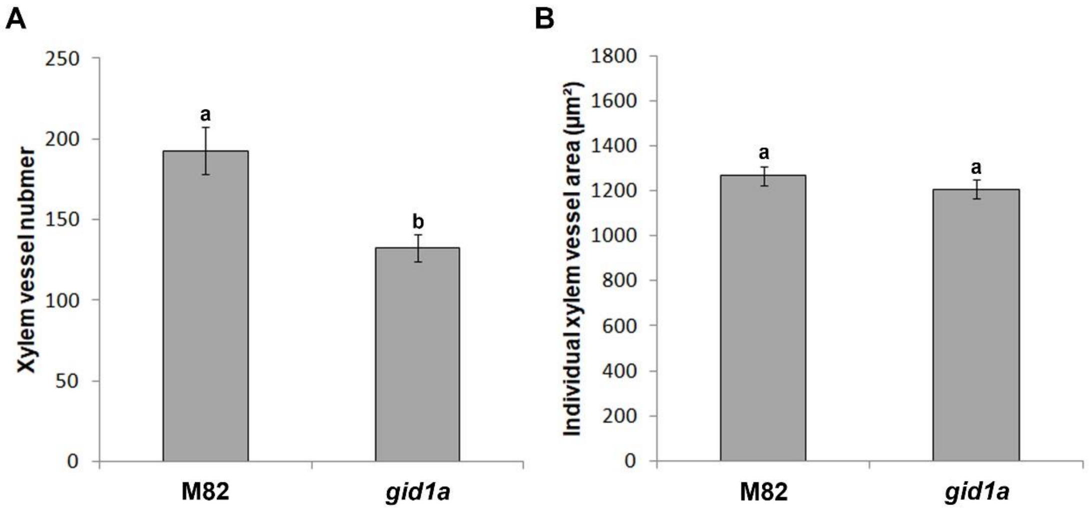

Fig. S2. A. Xylem vessel number in M82 and *gid1a* stems. **B.** Area mean of individual xylem vessel in M82 and *gid1a* stems. Values in **A** and **B** are means of 6 plants ± SE. Letters above the columns represent significant differences between respective lines (Student's t test, $P<0.05$).

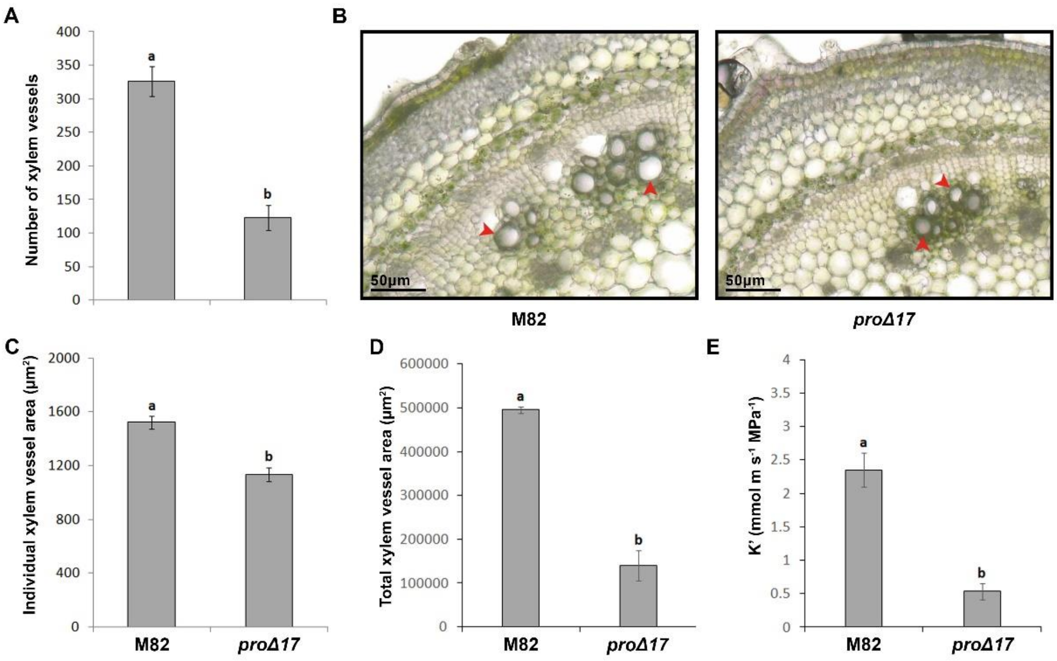

Fig. S3. A. Xylem vessel number in the stems of five-weeks-old M82 and *35:proΔ17* plants. **B.** Representative stem cross-sections of M82 and *35:proΔ17*. Scale bar = 50 μm. **C**. Area mean of individual xylem vessel in M82 and *35:proΔ17* stems**. D.** Total xylem vessel area in M82 and *35:proΔ17* stems. **E.** Hydraulic conductance measured in detached stem segments, taken from five-weeks-old M82 and *35:proΔ17*. Values in **A**, **C**, **D** and **E** are means of 6 plants ± SE. Small letters represent significant differences between the lines (Student's t test, P<0.05).

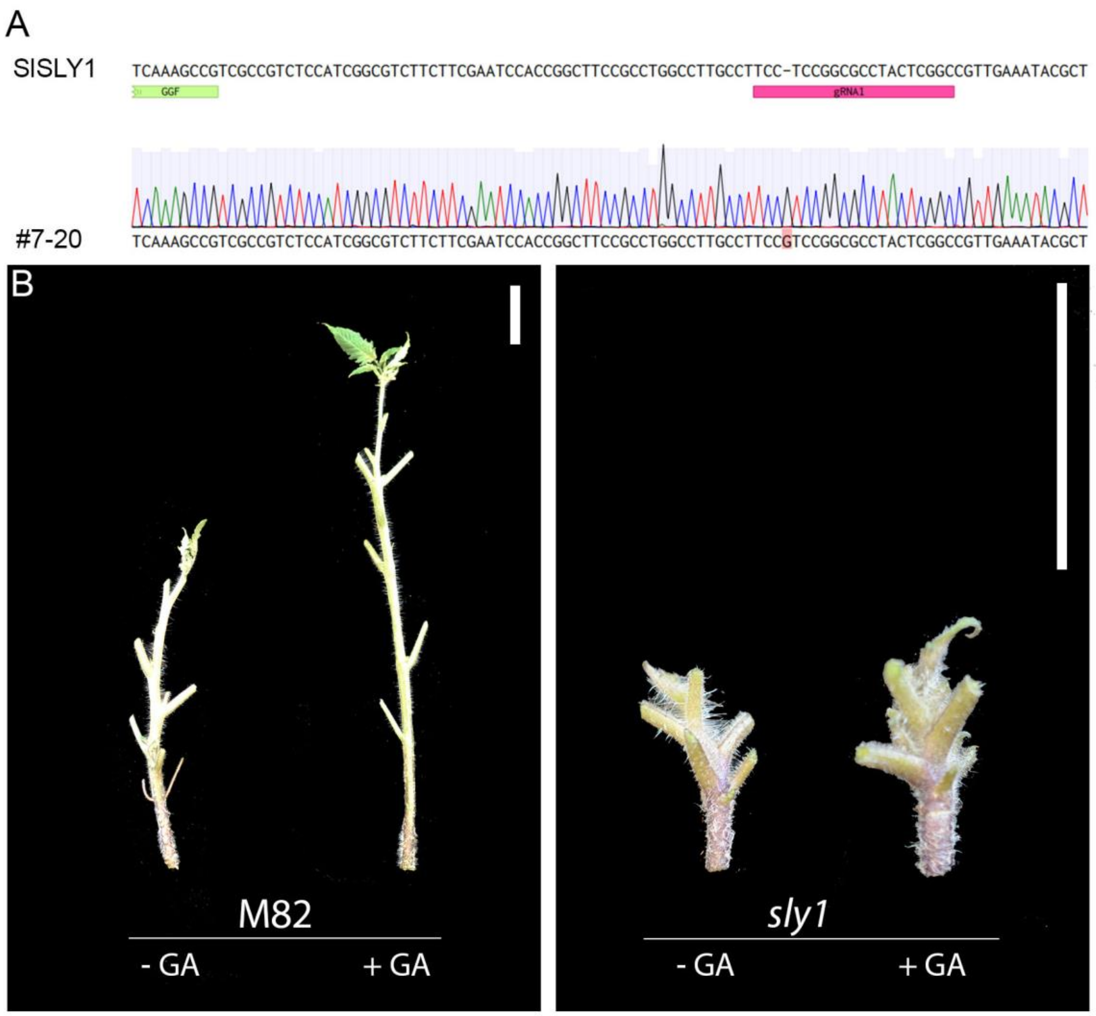

Fig. S4. Molecular and phenotypic characterization of *sly1*. Sequence of *SlSLY1* mutant alleles. **A.** Sequences of SLY1 wild-type (upper) and *sly1* mutant allele #7-20 with one nucleotide insertion (G, marked pink). Pink bar represents RNA guide sequence. **B**. Effect of 100 µM GA3 treatment on the elongation of M82 and *sly1*. Leaves were removed to show internode elongation. Scale bar = 2 cm.

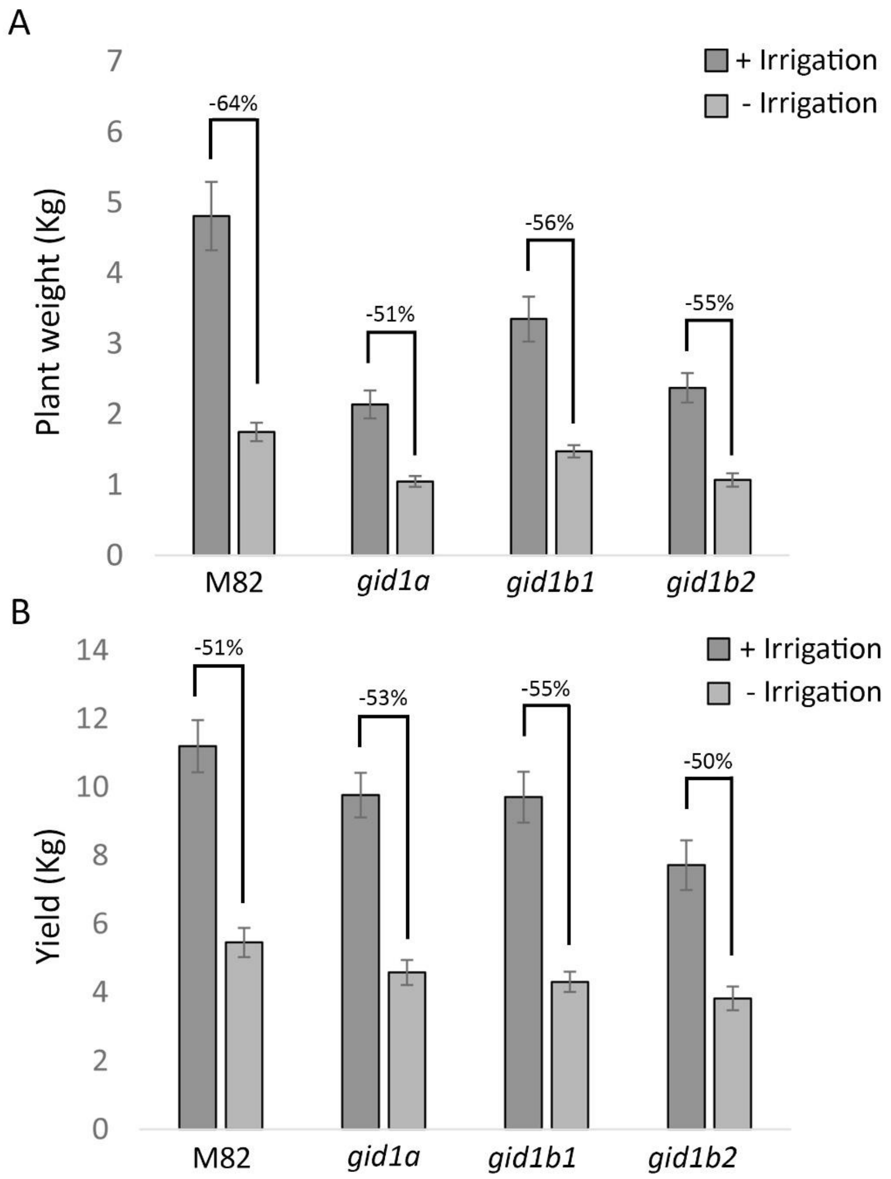

Fig. S5. Field test for yield loss under drought. M82 and all single *gid1* mutant were planted in the field. Plants from each line were planted randomly and were irrigated normally throughout the experiment. Half of the plants of each line were irrigated normally for three weeks and then irrigation was stopped until harvesting (approximately three more months). **A**. Reduction in plant weight under drought. **B**. Reduction in fruit yield under drought. Numbers above columns are percentage of loss.

Supplementary Table

Table S1. Primers used in this study.

Gene	Used for	Sequence (5'-3')
GID1a	qRT-PCR	Forward- TCTTGTTGTTGTCGCAGGTT<br>Reverse- CCCTATTGTTGCCTTCTCCA
GID1b1	qRT-PCR	Forward- GGCTGCTCTTCAATGGGTAA<br>Reverse- TAAAACCTCGACGCCTGATT
GID1b2	qRT-PCR	Forward- TGAGGCTGATTGGGGTAAAA<br>Reverse- TAGGCGGCGACAAAATGTAT
SlACTIN	qRT-PCR	Forward- GTCCTCTTCCAGCCATCCAT<br>Reverse- ACCACTGAGCACAATGTTACCG
SlSLY1	CRISPR/ Cas9	Guide 1- GCCGAGTAGGCGCCGGAGGA<br>Guide 2- GGTTATAGAGTCGCCGGATG
SlSLY1	Cloning to pBridge	Forward-<br>ATGAAGCGGCAATTCGAC<br>Reverse-<br>TTATTTATTAGCTTTGAAATTCATCTTCTCGTAG
